# Enhancement of the optical and electrical properties of poly ethyl methacrylate/polyvinyl chloride-zinc sulphide (PEMA/PVC@ZnS) ternary nanocomposite films

**DOI:** 10.1016/j.heliyon.2023.e21372

**Published:** 2023-10-21

**Authors:** Nuha Al-Harbi

**Affiliations:** Physics Department, Faculty of Applied Sciences, Umm Al-Qura University, Makkah, Saudi Arabia

**Keywords:** PEMA/PVC blend, PEMA/PVC@ZnS nanocomposites, FT-IR, X-ray, UV–Visible spectroscopy, Dielectric parameters

## Abstract

Our research introduces a novel ternary nanocomposite consisting of polyethyl methacrylate/polyvinyl chloride-Zinc sulphide nanoparticles (PEMA/PVC@ZnS). Zinc sulphide (ZnS) nanoparticles were produced via a chemical method and then dispersed at different concentrations (0.02, 0.05, 0.08, and 0.1 wt%) in a single step within the PEMA/PVC blend. The resulting PEMA/PVC@ZnS nanocomposite films were analyzed to investigate their spectroscopic and electrical properties. The dielectric parameters of the samples were also studied in detail. X-ray diffraction (XRD) data indicated an increase in the amorphous region and demonstrated the interaction between ZnS and PEMA/PVC. Fourier transform infrared (FT-IR) results confirmed the specific interactions in PEMA/PVC@ZnS nanocomposites. The synthesized films showed a distinct absorption band at 432 nm, which was attributed to the ZnS surface plasmon resonance. As the concentration of ZnS in PEMA/PVC increased, the band gap energies decreased for both direct and forbidden transitions. Optical parameters such as the extinction coefficient (k), refractive index (n), dielectric constants (*ε*′ and *ε*''), optical conductivity (σ_(opt._), and **photoluminescence (PL)** were also studied. The values of dielectric permittivity and dielectric modulus from AC measurement of PEMA/PVC@ZnS nanocomposite films increased with increasing ZnS content. The data suggest that PEMA/PVC@ZnS nanocomposite films exhibit excellent optical and electronic properties, making them suitable for use in various electric and optoelectric applications.

## 1 Introduction

Poly ethyl methacrylate (PEMA) is a copolymer that combines ethylene and methacrylic monomers [1,2]. PEMA is known for its excellent adhesive properties and is commonly used in producing pressure-sensitive adhesive films and tapes. The presence of the methacrylic units provides carboxylic functional groups that enhance its adhesion to a wide range of substrates [3,4].

The wide use of polyvinyl chloride (PVC) as an industrial polymer in various applications is attributed to its durability, versatility, and low cost [5,6]. PVC is a widely used polymer with multiple applications, but its environmental impact should be considered when evaluating its use. Efforts are being made to develop more sustainable alternatives to PVC in various industries [7,8].

A blend of PEMA and PVC polymers has some beneficial properties and applications. PEMA and PVC are miscible polymers, meaning they can form homogeneous blends at the molecular level. This results in a material with properties that combine the two polymers. The PEMA/PVC blend exhibits improved flexibility compared to pure PVC. PVC is typically relatively rigid, while PEMA is more flexible. The blend has good thermal stability. The ratio of PEMA to PVC can be varied to control the properties of the blend [9,10]. Higher PEMA content results in greater flexibility, impact strength, and transparency at the cost of lower heat resistance.

Polymers containing nanoparticles have been extensively researched in recent years due to their multiple properties and potential for critical scientific applications in various fields [11]. Incorporating nanoparticles into polymer matrices can produce materials with enhanced mechanical, electrical, thermal, and optical properties for potential applications, including electronics, optics, and engineering applications [12,13].

Zinc sulphide (ZnS) nanoparticles are an inorganic compound. ZnS nanoparticles exhibit strong quantum confinement effects [14,15] and size-tunable photoluminescence [16,17]. The dispersion and interaction of the ZnS nanoparticles within the PEMA/PVC matrix influence the extent of property improvement. Surface modification of the nanoparticles can help enhance their compatibility and dispersion.

Moreover, the literature on PEMA/PVC-ZnS is limited, highlighting the necessity of developing a novel polymer nanocomposite material incorporating ZnS nanoparticles into a PEMA/PVC blend matrix. The literature suggests that ZnS nanoparticles are promising nanofillers for producing PEMA/PVC polymer nanocomposites with enhanced mechanical, thermal, optical, and electrical properties compared to the base PEMA/PVC blend. Maheswari et al. (2018) performed the FT-IR analysis of PEMA/PVC blend doped ZnS nanocomposites [18]. Maruthan et al. (2019) performed UV–Vis absorption spectroscopy of the nanocomposites [19]. They observed that all nanocomposites showed an absorption edge below 400 nm attributed to the band gap absorption of ZnS nanoparticles. The study by Lin and colleagues (2014) reported the synthesis of ZnS nanoparticles and their dispersion in a PEMA/PVC blend matrix [20]. The resulting nanocomposite exhibited improved UV-blocking properties. Similarly, a study by Zhao and colleagues (2019) reported the synthesis of PEMA/PVC/ZnS nanocomposites by solution blending, which exhibited improved mechanical and thermal properties [21]. The present PEMA/PVC-Zns used a different ratio between PEMA and PVC incorporated with a different ratio of Zns nanoparticles, leading to material properties and performance variations. The present system might also involve unique preparation techniques or modifications that enhance its structural and functional aspects. These differences contribute to the uniqueness and potential advantages of the present system compared to the previously published systems.

The primary goal of this study is to synthesize PEMA/PVC@ZnS nanocomposites using casting solution methods. Subsequently, the spectroscopic, optical, and electrical properties of the nanocomposites will be investigated and characterized using a suite of advanced analytical techniques, including X-ray diffraction (XRD), Fourier transforms infrared (FT-IR) spectroscopy, UV–Visible (UV–Vis) spectroscopy, and electrical measurements. By elucidating the fundamental properties of PEMA/PVC@ZnS nanocomposites, these films showcase exceptional optical and electronic properties, underscoring their immense potential for diverse and cutting-edge applications in the fields of electronics and optoelectronics.

## 2 Experimental work

### 2.1 Materials

Polyethyl methacrylate (PEMA, C_6_H_10_O_2_) with an average molecular weight of ∼515000, polyvinyl chloride (PVC, C_2_H_3_Cl), and acetone were procured from Sigma-Aldrich.

### 2.2 Preparation of pure ZnS nanoparticles

To prepare Zinc sulphide (ZnS) nanoparticles, an equimolar mixture of 25 mL of zinc acetate (ZnC₄H₆O₄) with (0.5 M) thioacetamide (C_2_H_5_NS) were precursors for the formation of ZnS nanoparticles. The mixture solution ZnC₄H₆O₄ and C_2_H_5_NS was stirred continuously under magnetic stirring to achieve a uniform distribution for about 3 h at 85 °C to enable the reaction and lead to forming ZnS nanoparticles. After the heating step, the mixture was left to cool to room temperature. Then, collect the ZnS nanoparticles by centrifugation, which helps separate the nanoparticles from the remaining liquid. The collected ZnS nanoparticles were washed several times with absolute ethanol (C_2_H_5_OH) to remove impurities. Finally, the collected ZnS nanoparticles were dried for 12 h in a dryer oven at 60 °C to completely remove any remaining solvent.

### 2.3 Preparation of PEMA/PVC@ZnS nanocomposites

The PEMA/PVC@ZnS nanocomposite films were synthesized using the solution casting method. In a typical experiment, 4 g of pure PEMA polymer were dissolved in 40 mL pure acetone, and 2 g pure PVC polymer were dissolved in 20 mL pure acetone. These individual solutions were stirred in an oven at 48 °C for about 4 h to ensure complete dissolution and homogeneity. The dispersion of different concentrations of ZnS nanoparticles (0, 0.02, 0.05, 0.08, and 0.1) was added drop by drop to the PEMA/PVC blend solutions under sonication for a period of about 20 min to ensure that the nanoparticles were uniformly dispersed without any indication of sedimentation. These homogeneous solutions were poured onto Petri dishes and left to dry. The thickness of the nanocomposite films ranged from ∼200 μm. A schematic diagram 1 illustrates the method for preparing PEMA/PVC@ZnS nanocomposite films. A schematic diagram 2 shows the possible chemical interaction between PEMA, PVA, and ZnS nanocomposites.

**A schematic 1:**
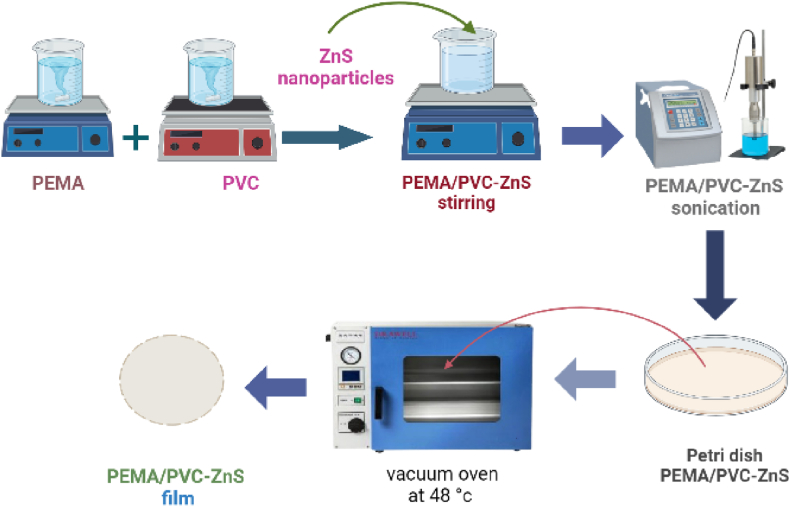
The method for preparing and blending PEMA/PVC incorporated with ZnS nanoparticles.

**Schematic 2:**
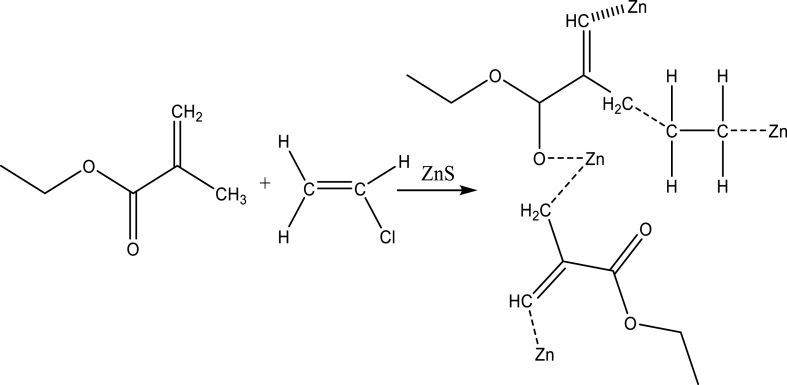
Possible of chemical interaction between PEMA, PVA, and ZnS nanocomposites.

### 2.4 Characterization

The structure and crystalline size of the PEMA/PVC@ZnS nanocomposite films were analyzed using X-ray diffraction (XRD) by a BRUKER D8 ADVANCE diffractometer. Fourier-transform infrared (FT-IR) spectra were using an ATR mode Nicolet UR 200 FT-IR spectrometer in the wavenumber 4000-400 cm-1 range. Transmission Electron Microscope (TEM) operating at 30 kV was used to examine the particle morphology and particle size of ZnS nanoparticles using an FEI Tecnai G2. UV–visible spectra were measured using (a JASCO, Japan-type V-570) double beam spectrophotometer in the range of wavelength 190–1000 nm. Photoluminescence data were recorded in the range of frequency from 400 to 900 nm with excitation of 360 nm. The AC measurements were obtained using A high-resolution Alpha-A Analyzer, manufactured by Novocontrol within the frequency range of 0.1 Hz–6 MHz.

## 3 Results and discussion

### 3.1 X-ray diffraction

**Fig. 1:**
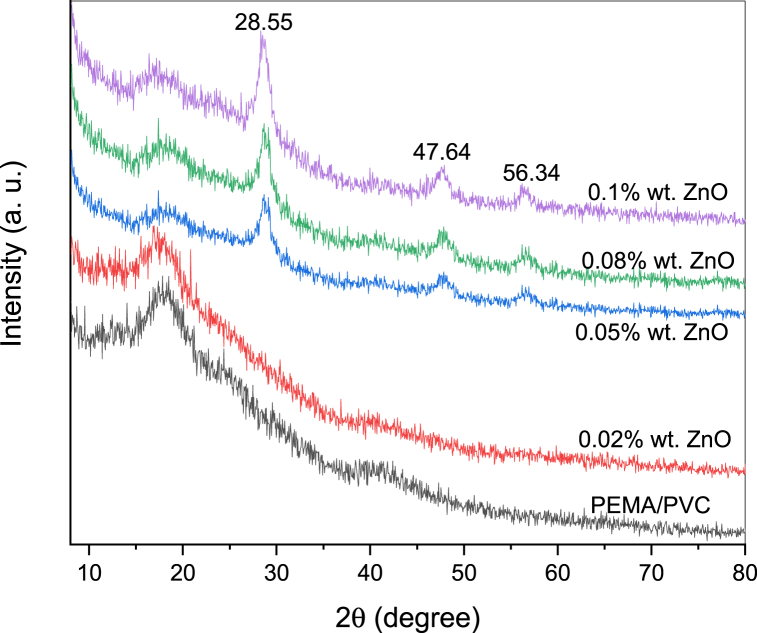
X-ray diffraction (XRD) pattern of PEMA/PVC incorporated with ZnS nanoparticles.

Fig. 1 displays (inserted) the X-ray (XRD) diffraction spectrum of pure Zinc sulphide (ZnS) nanoparticles. The information from the spectrum suggests that the XRD of ZnS nanoparticles exhibit three diffraction peaks at 2θ values of 28.5°, 47.4°, and 56.3°. These peaks are related to the (111), (220), and (311) planes [[22], [23], [24]], respectively; these peaks match perfectly with the corresponding planes reported in the JCPDS number 05–0566, indicating that the ZnS nanoparticles have a crystalline structure that is consistent with the face-centred cubic structure of ZnS. Due to the small crystal size, the peaks of the samples exhibit broadness. Additionally, the absence of diffraction peaks from impurities was noted. The lattice parameter of 5.41 Å and the cell volume of 158.4 Å, as in ICDD PDF 65–1691, provide additional information about the crystal structure of ZnS. The lattice parameter is the distance between adjacent lattice points in the crystal lattice, while the cell volume is the volume of the unit cell that contains one or more lattice points.

For pure PEMA, the XRD spectrum typically shows a diffraction of two peaks at around 2θ = 11.65° and 19.5°, which corresponds to the (200) and (110) plane of the crystal lattice [25,26]. These peaks are broad and asymmetric, which indicates that the polymer chains are not perfectly ordered in the crystal lattice and that there are some defects in the arrangement. These broad Bragg's peaks indicate the amorphous nature of the PEMA. Whereas the XRD pattern of PVC typically shows sharp peaks at around 2θ = 17.7° and 24.5° [27], which correspond to the (200), and (210) planes of the semi-crystalline phase, respectively. This indicates that the PVC has both crystalline and amorphous regions.

The X-ray spectra demonstrate a decrease in the intensity of PEMA/PVC, ascribed to the interaction between ZnS that occurs in the amorphous regions within PEMA/PVC chains. The main peaks of ZnS are observed in the spectra of PEMA/PVC@ZnS nanocomposites. The samples exhibited an absence of diffraction peaks associated with impurities. There are no X-ray peaks at a low concentration (0.02 wt%) of ZnS due to the low concentration of ZnS within the polymer blend.

The Scherrer equation estimates the average size of ZnS nanoparticles embedded within PEMA/PVC. The Scherrer equation is given by Ref. [26]:

(1) $$D=\frac{k\lambda }{\beta \;\cos\;\theta }$$

where: D represents the average crystallite size, *K* is the Scherrer constant, which depends on the shape of the crystallite (typically assumed to be around 0.9), λ is the wavelength of the X-ray radiation used for the measurement, β is the full width at half maximum (FWHM) of the diffraction peak related to the crystal lattice plane, and θ is the Bragg angle. The FWHM of these peaks can be measured, representing the peak's angular width at half of its maximum intensity. The broader the FWHM, the smaller the crystallite size. The Bragg angle, θ, can be determined based on the crystal structure of ZnS and the particle size [28,29]. The average particle size, D, can be calculated by substituting the values of K, λ, β, and θ in the 16 nm range. Other techniques, such as transmission electron microscopy (TEM) can be employed to obtain more detailed information about the nanoparticles' particle size distribution and morphology.

### 3.2 FT-IR study

**Fig. 2:**
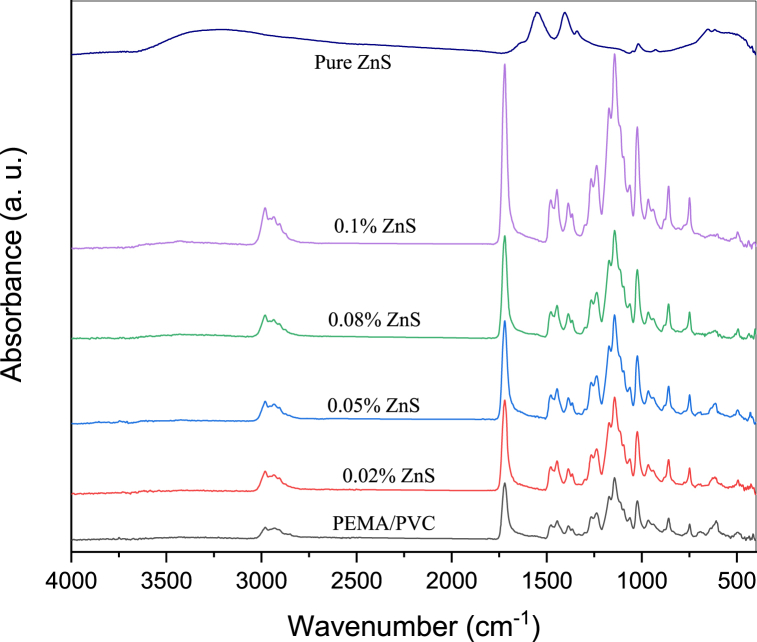
FT-IR spectra of PEMA/PVC incorporated with ZnS nanoparticles.

Fig. 2 illustrates the FT-IR spectra of the PEMA/PVC and PEMA/PVC incorporated with different weight percentages of ZnS. In pure PEMA, several IR bands are observed, including the 1024 cm^−1^ band due to C–C stretch, 939 cm^−1^ band due to C–O stretch, 859 cm^−1^ band due to C–H bend, 748 cm^−1^ band due to C–H bend, and 494 cm^−1^ band for C–H bend. The spectrum of pure PEMA also displayed bands at 2981 cm^−1^ and 2936 cm^−1^, which are related to O–H stretching and asymmetric C–H stretching. The bands at 1732 cm^−1^ and 1478 cm^−1^ are due to the C=C stretching band of an unconjugated ester (free carbonyl group) and CH_2_ scissoring δ(CH_2_), while the peak at 1446 cm^−1^ is characteristic of the O–CH_3_ ester group. The band at 1387 cm^−1^ is assigned to out-of-phase bending vibrations of the CH_3_ [[30], [31], [32], [33]].

The FT-IR spectrum of PVC displays two distinct bands at 2970 cm^−1^ and 2913 cm^−1^ for the CH_2_ asymmetric stretching vibration mode. The spectrum also exhibits a modification of the OH band at 3400 cm^−1^, and a weak shoulder at 1646 cm^−1^, representing the C=O stretching vibration. The band at higher wavenumbers corresponds to the asymmetric stretching bond of C–H, while the lower band is for the symmetrical stretching bond of C–H. The bands corresponding to the C–H stretching of the methylene groups appear at 1425 cm^−1^ and 975 cm^−1^. The CH_2_ twisting deformation modes observed at 1330 cm^−1^, and the band at 1252 cm^−1^ are related to the bending bond of C–H. The C–C stretching bond of the PVC backbone chain occurs in the range of 1095 cm^−1^. Finally, bands at 689 cm^−1^ and 610 cm^−1^ are assigned to the C–Cl gauche bond [34,35].

The IR spectrum of the PEMA/PVC blend exhibits changes in the spectral features compared to the individual spectra of PEMA and PVC. The changes in the IR spectrum suggest the occurrence of complexation between PEMA and PVC polymers.

PEMA and PVC are two polymers that can be blended to create a material with unique properties. When PEMA and PVC are blended, several interactions can occur between the polymer chains. One type of interaction that can occur is hydrogen bonding. PEMA contains polar groups such as carbonyl and ester groups, which can form hydrogen bonds with the chloride atoms in PVC. Another type of interaction that can occur is van der Waals forces between the PVC and PEMA chains can cause them to attract each other, improving the blend's miscibility.

In the IR spectrum of the PEMA/PVC blend, the characteristic bands of the individual components of PEMA and PVC are still present, and other bands are disappeared, especially for PVC. The bands at 1431 cm^−1^ and 1329 cm^−1^, which correspond to the O–CH_3_ ester group and out-of-phase of the CH_3_, respectively, show a decrease in intensity.

The IR spectrum of pure ZnS nanoparticles exhibits an intense band observed around 3427 cm^−1^ due to the O–H stretching modes. The band at 2923 cm^−1^ may arise from the microstructure formation of pristine ZnS. The bands observed at 1416 and 1585 cm^−1^ correspond to the stretching modes of carboxyl (C=O) groups. The sharp adsorption band centred at 998 cm^−1^ is caused by the resonance interaction between the vibrational modes of sulfide ions in the ZnS crystal. The absorption bands at 495 and 626 cm^−1^ are attributed to Zn–S vibration.

The FT- IR spectra of PEMA/PVC doped with different concentration of ZnS nanoparticles exhibits changes compared to the spectra of the undoped polymer blend. The changes in the FT-IR spectra suggest the occurrence of interactions between the nanoparticles and the polymer matrix. In the spectrum of the doped blend, the characteristic bands of PEMA and PVC are still present but with some shifts and intensity changes. The most significant changes in the spectra of the doped blend occur in the region of the ZnS nanoparticles' characteristic absorption bands. Most bands, such as Zn–S stretching and the Zn–S–H bending vibration, are observed, and most bands become more prominent in the doped blend spectrum. These observations suggest that the ZnS nanoparticles are interacting with the polymer molecules.

### 3.3 TEM

**Fig. 3:**
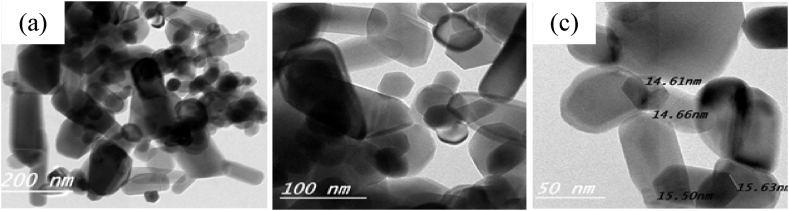
The TEM image of pure ZnS particles at different magnifications; (a) 200 nm, b) 100 nm, and c) 50 nm.

The TEM image of the ZnS particles presented at different magnifications (200, 100, and 50 nm) in Fig. 3 a-c revealed almost rods and semi-spherical aggregates composed of nanometer-sized (∼14.6±2 nm) crystals. This observation agrees with the XRD data that showed broad diffraction peaks, indicating the small size of the crystals. The observed aggregation of the nanoparticles is likely due to the formation of S–S bridges between sulfide particles. These bridges can form due to the high reactivity of the surface sulfide groups, which can react with each other to form disulfide bonds. The formation of these S–S bridges can lead to the aggregation of the nanoparticles, resulting in the observed morphology.

### 3.4 Optical study

**Fig. 4:**
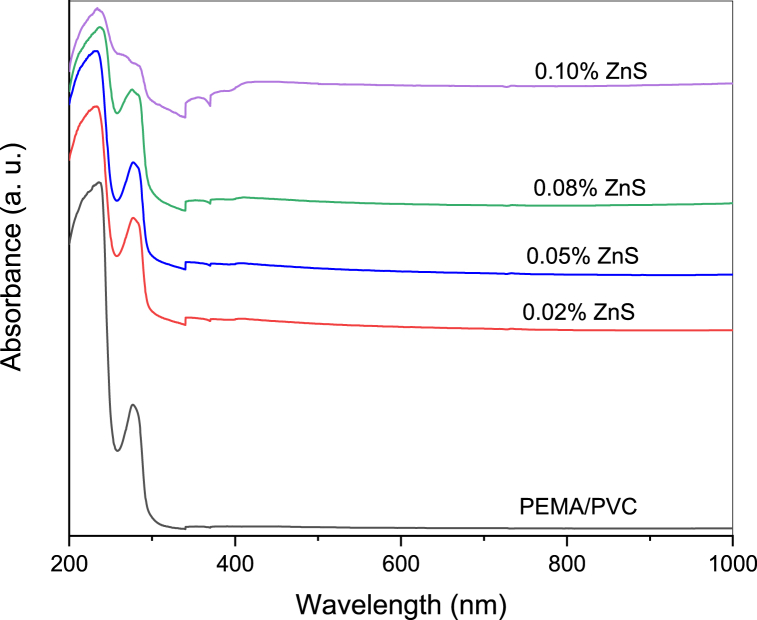
UV–visible absorption spectra of PEMA/PVC incorporated with ZnS nanoparticles.

Fig. 4 illustrates the absorbance spectra of PEMA/PVC composite materials that have been incorporated with varying concentrations of ZnS. The transparent nature of PEMA and PVC results in minimal absorption within the visible region. Thus, the PEMA/PVC blend is expected to exhibit transparency and low absorbance within the visible range. The absorbance spectrum of PEMA/PVC shows an absorption band in the UV region at wavelength *λ* = 233 nm and a strong absorbance band at λ = 280, which can be assigned to the π-π*and n-π* transition.

The absorption coefficient (α) of produced films can be determined from the absorption (A) and thickness (d) using the Beer-Lambert law [36,37]:

(2) $$\alpha =\frac{2.303}{d}\;A$$

**Fig. 5:**
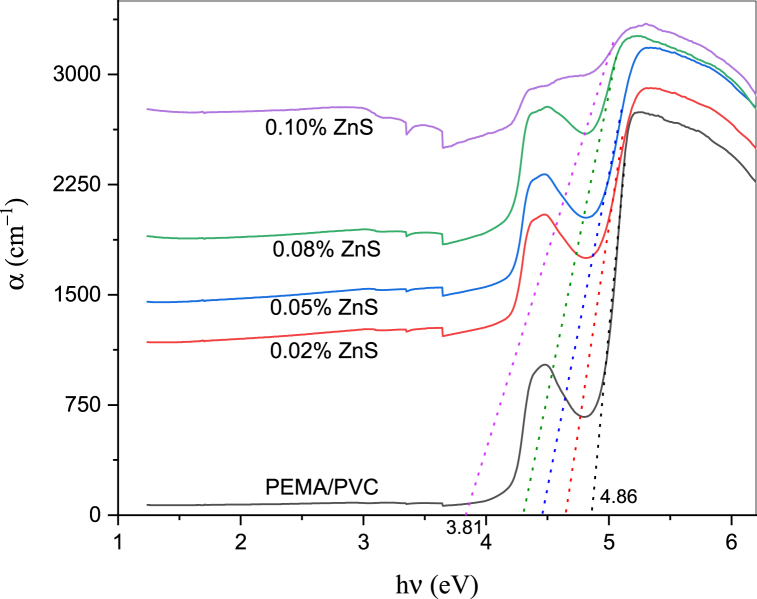
Relationship between absorption coefficient (α) and photon energy (hν) of PEMA/PVC incorporated with ZnS nanoparticles.

The changes in the absorption coefficient (α) spectra as a function of photon energy (h***&upsi***) are shown in Fig. 5. By extrapolating the straight line segment of the curve to the point where α = 0, we can determine the value of the absorption edge. The absorption edge (E_d_) values are presented in Table 1. It can be observed that there is a red shift in the absorption edge, which is measured to be 4.86 eV for PEMA/PVC. However, the absorption edge gradually decreased in the case of doped films, with the value reaching 3.81 eV for PEMA/PVC-0.1 % ZnS film.

**Table 1:** The values of absorption edge (E_d_), direct (E_d_), direct allowed ($E_{g}^{d}$), and direct forbidden ($E_{g}^{\text{in}}$) optical band gap energies of PEMA/PVC@ZnS nanocomposites.

Samples	absorption edge (E_d_)	direct allowed band gap ($E_{g}^{d}$)	direct forbidden band gap ($E_{g}^{\text{in}}$)
PEMA/PVC	4.86	4.96	4.99
0.02 % ZnS	4.64	4.89	4.88
0.05 % ZnS	4.44	4.79	4.83
0.08 % ZnS	4.28	4.67	4.76
0.10 % ZnS	3.81	4.33	3.53

The optical absorption spectra are a valuable tool for determining the optical band gap of materials. Davis and Mott reported a correlation that indicates the absorption coefficient (α) on the photon energy hυ as the following [38]:

(3) $${\left(\alpha h\upsilon \right)}^{2}=A\left(h\upsilon -E_{g}^{d}\right)\;\text{for}\;\text{direct}\;\text{allowed}\;\text{transition}$$

(4) $${\left(\alpha h\upsilon \right)}^{2/3}=A\left(h\upsilon -E_{g}^{\text{in}}\right)\;\text{for}\;\text{direct}\;\text{forbidden}\;\text{transition}$$

**Fig. 6:**
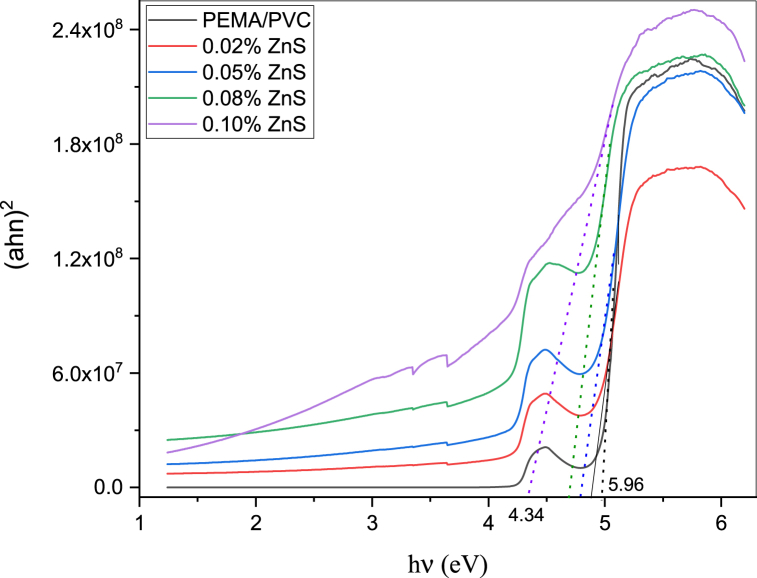
Relationship between (αhν)^2^ and photon energy (hν) of PEMA/PVC incorporated with ZnS nanoparticles.

**Fig. 7:**
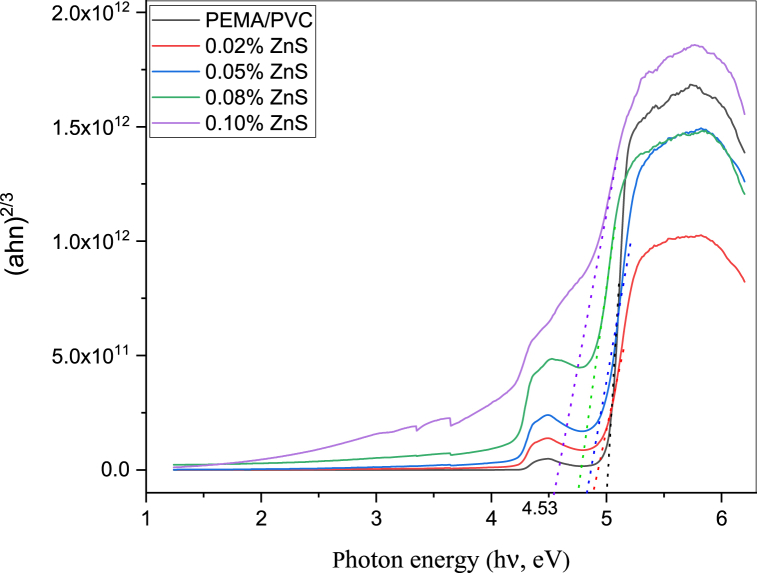
Relationship between (ahn)^2/3^ and photon energy (hν) of PEMA/PVC incorporated with ZnS nanoparticles.

Where A is a constant, h is Planck's constant, and υ is frequency. The direct and direct forbidden optical energy gap values are estimated by plotting the relation between ${\left(\alpha h\upsilon \right)}^{2}$ and ${\left(\alpha h\upsilon \right)}^{2/3}$ against photon energy (E = hυ), as shown in Fig. 6, Fig. 7. Where the extrapolation of the linear portion of the curves to a point ${\left(\alpha h\upsilon \right)}^{2}$ and ${\left(\alpha h\upsilon \right)}^{\frac{1}{2}}$ = zero gives the values of both direct allowed $E_{g}^{d}$ and direct forbidden $E_{g}^{\text{in}}$ band gap energy as we see in Table 1. It has been determined that the material exhibits a reduction in both its direct allowed and direct forbidden energy band gaps.

From the data, adding ZnS nanoparticles to PEMA/PVC chains decreases the band gap energy values. The interaction/coordination leads to the generation of localized states within the band gap. The reduction in E_d_, $E_{g}^{d,}$ and $E_{g}^{f}$ can be explained by the improvement in the degree of disorder within the filled samples. The addition of ZnS induces expanding of the localized energy levels, which leads to an increase in the degree of disorder. This, in turn, contributes to the decrease in the bandgap energies.

The extinction coefficient (k) characterizes how a material interacts with the light of a certain wavelength and reveals the variations in absorption that occur as the electromagnetic wave travels through the material. It is mathematically related to the absorption coefficient through the following equation [39]:

(5) $$K=\frac{\alpha \lambda }{4\pi }$$

**Fig. 8:**
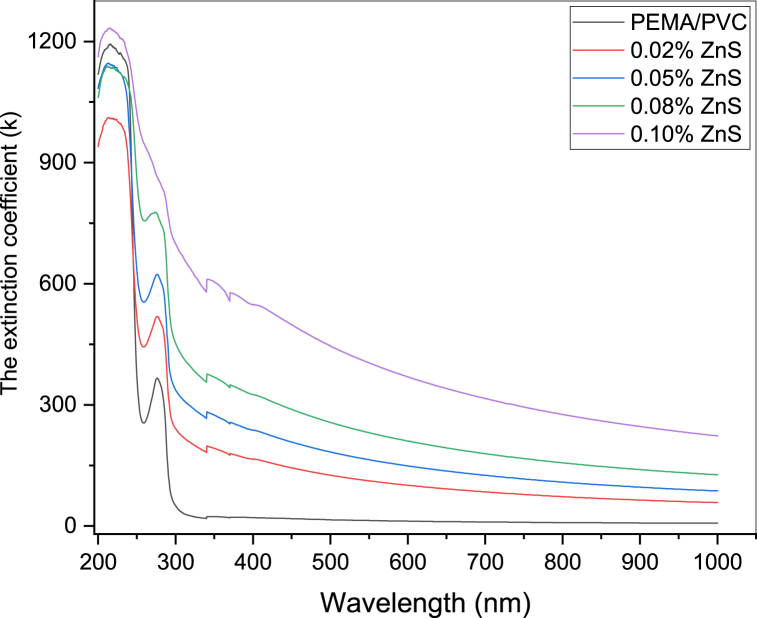
Relationship between the extinction coefficient (k) and wavelength (λ) of PEMA/PVC incorporated with ZnS nanoparticles.

Fig. 8 displays the plot between the extinction coefficient (k) and the photon energy (E = hν). As depicted in the figure, the extinction coefficient increases with increasing levels of ZnS doping in the PEMA/PVA polymer matrices), indicating greater light scattering and absorption by the ZnS particles. This increase in k is directly proportional to the absorption coefficient, indicating a corresponding rise in the absorption characteristics. Furthermore, the higher ZnS content in the PEMA/PVC matrix at longer wavelengths leads to increased photon scattering, thereby contributing to the increased extinction coefficient.

The complex refractive index (n) of the samples is calculated from the reflectance (R) as [40]:

(6) $$n=\frac{1+R}{1-R}+\sqrt{\frac{4R}{{\left(1-R\right)}^{2}}-k^{2}}$$

The refractive index of PEMA/PVC@Zns nanocomposite samples is estimated (between 1.742 for pure PEMA/PVC to 1.761 for PEMA/PVC doped with 0.1 % wt. of ZnS) to be higher than that of the pure PEMA/PVC blend. This feature is of fundamental importance in all conductors, as it arises from the centralized alteration of charged particles within the medium.

There is a relationship between the refractive index (n) and the energy gap of a material. The energy gap is the difference between the highest occupied energy level (valence band) and the lowest unoccupied energy level (conduction band). The refractive index measures how much the speed of light is slowed down when it passes through the sample. So, a higher refractive index corresponds to a lower energy gap because the samples with lower energy gaps tend to have stronger interactions with light, causing a greater slowing down of light and a higher refractive index.

The dielectric constants were analyzed, revealing variations in the real (*ε*′) and imaginary (*ε*'') parts. It was observed that the real part (*ε*′) of the dielectric constant is dependent on the refractive index (n), which explains the insignificantly small extinction coefficient (k) result that may be disregarded in conformity with the relevant equation [[39], [40], [41]]:

(7) $$\varepsilon^{\prime }=n^{2}-K^{2}$$

The dielectric imaginary part (*ε*'') is calculated as a component of the dielectric constant using the equation [[39], [40], [41]]:

(8) $$\varepsilon^{\prime \prime }=2\text{nK}$$

The calculated values of real (*ε*′) and imaginary (*ε*'') parts of the dielectric constant increase in PEMA/PVC@Zns nanocomposite more than in pure PEMA/PVC polymer blend. The observation implies that the samples exhibit distinct structures. Therefore, changes in doping conditions lead to variations in the chemical composition of the polymer.

Optical conductivity ($\sigma_{\text{opt}.}$) denotes an optical parameter that provides information about the electronic configuration of the material under study. Optical parameters absorption coefficient (α) and refractive index (n) have been used as indicators from which the value of optical conductivity ($\sigma_{opt.}$) may be derived from the relation [39,40].

(9) $$\sigma_{Opt.}=\frac{\lambda nc}{2\pi }$$

**Fig. 9:**
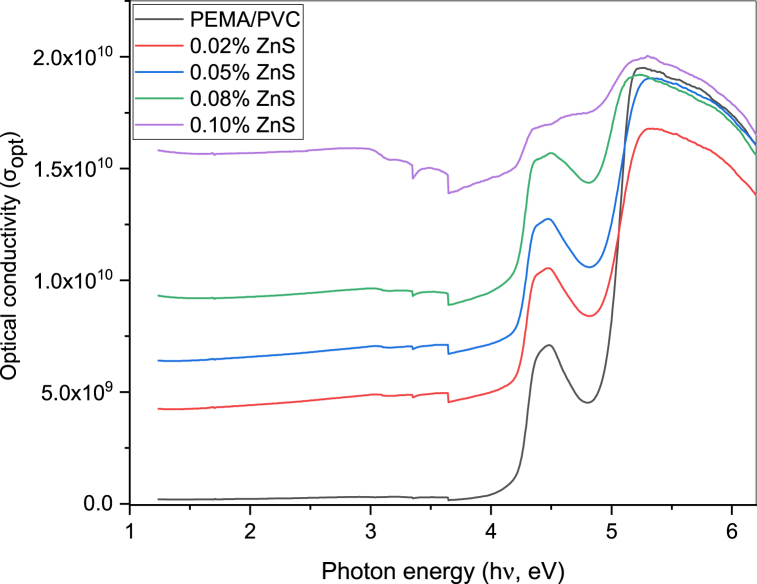
Relationship between the optical conductivity ($\sigma_{\text{opt}.}$) and the photon energy (hν) of PEMA/PVC incorporated with ZnS nanoparticles.

The variation in $\sigma_{\text{opt}.}$ against the photon energy (hν) for the samples are shown in Fig. 9. The estimated value of $\sigma_{\text{opt}.}$ tends to increase at higher energies due to larger values of n and α. As (hν) and ZnS contents increase, the values of $\sigma_{\text{opt}.}$ Also, increase in PEMA/PVC@ZnS nanocomposite samples. The increase in $\sigma_{\text{opt}.}$ at high energies could be due to the high absorption through these samples. The data shows that the optical conductivity increases significantly near the absorption edge. This phenomenon is attributed to the surplus of free charge carriers within the nanocomposite films, enabling it to surmount its intrinsic bandgap through optical transitions that would otherwise be forbidden. Excess free electrons and holes act as additional pathways for photon absorption, resulting in a steep increase in optical conductivity near the absorption edge wavelength. The free charge carriers allow optical transitions between previously energetically forbidden states, manifesting as a rise in the measured optical conductivity and indicating that the band gap of the polymer film has been overcome. The upsurge in optical conductivity is thus due to the surplus of free charge carriers that provide more electronic states for photon absorption, crossing the band gap of the samples.

### 3.5 Photoluminescence (PL) measurements

**Fig. 10:**
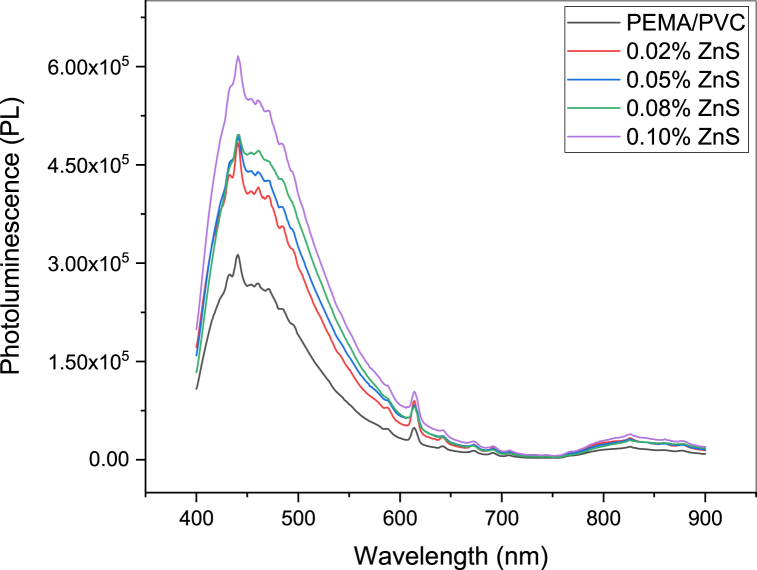
Photoluminescence (PL) spectra of PEMA/PVC doped with ZnS.

Photoluminescence can further support UV–visible measurements and the interaction between ZnS and 10.13039/100004899PEMA/PVC blend. Fig. 10 depicts the PL spectra of PEMA/PVC incorporated with different concentrations of ZnS nanocomposite films in the range of frequency from 400 to 900 nm at room temperature, excited with a wavelength of 360 nm. Pure PEMA/PVC displays less significant PL sensitivity, while a strong emission peak at 442 nm, exhibiting a clear excitonic emission feature, is observed in PEMA/PVC@ZnS nanocomposite films [42,43]. This finding provides compelling evidence for the interaction between ZnS and PEMA/PVC blend. It was found that when excited, the films emit light in the visible range from 400 to 580 nm. The emission increase originates from the sulfur vacancies located at the surface of ZnS.

### 3.6 Electrical conductivity

#### 3.6.1 The dielectric permittivity

**Fig. 11:**
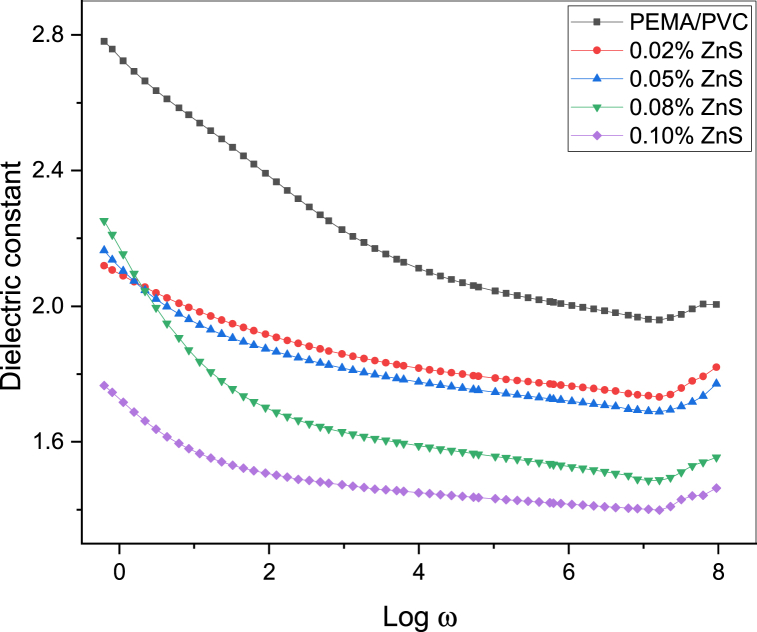
The plot between the dielectric constant and log w of PEMA/PVC doped with ZnS.

**Fig. 12:**
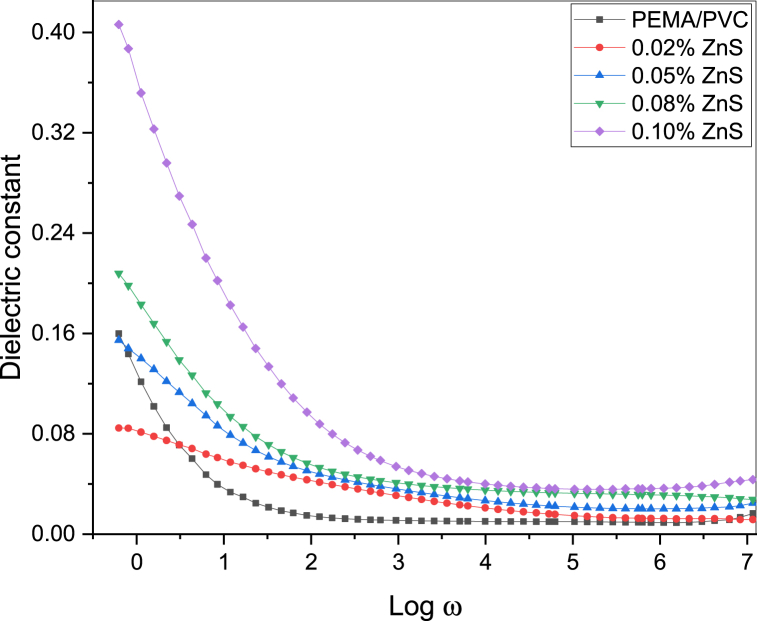
The plot between the dielectric loss and log ω of PEMA/PVC doped with ZnS.

**Fig. 13:**
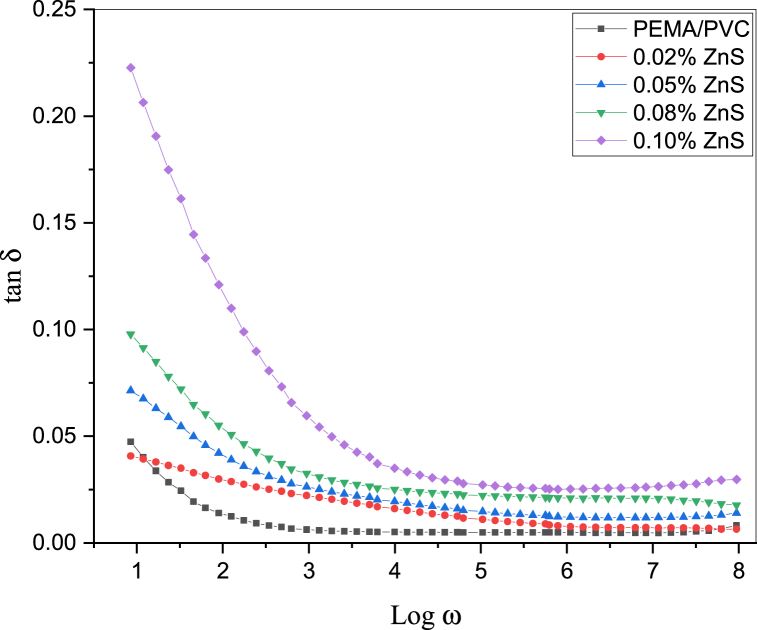
The plot between the loss tangent (tanδ) and log w of PEMA/PVC doped with ZnS.

Fig. 11, Fig. 12, Fig. 13 illustrate the frequency-dependent behavior of the dielectric constant (real part) and dielectric loss (imaginary part) of complex permittivity, as well as the dielectric loss tangent (tanδ) spectra, for PEMA/PVC@ZnS nanocomposite films as a function of the frequency (w = 2πf) in the range of frequency from 0.1 Hz to 6 MHz.

Notably, the values of real and imaginary parts of ZnS doped polymer blend films are higher than those of pristine PEMA/PVC (75/25) blended films. As we see in Fig. 11, the values of the real part serve as a measure of a material's ability to store electrical energy, and it is observed that the real part values of the prepared films exhibit a decrease with an increase in frequency throughout the frequency range. However, the rise in the real part values occurs gradually with a decrease in frequency within the frequency region. The observed increase in the real part values at low frequencies can be explained by the dominant contribution of the interfacial polarization effect, commonly referred to as the Maxwell-Wagner-Sillars effect. An increase in ZnS nanoparticle content results in a corresponding increase in the dielectric constant. This behavior is attributed to the interactions between the inorganic nanoparticles (ZnS) and the functional polar groups present in the polymer blend, which lead to the arrangement of dipoles.

Fig. 12, Fig. 13 demonstrated that the dielectric loss and dielectric loss tangent (tanδ) exhibited relatively higher values at lower frequencies, which decreased with increasing frequency [40] Interface space charge polarization, which has a lower relaxation frequency, played a significant role in these nanocomposites. Polymer composites contain two types of charge carriers: polaron or/and bipolaron contribution from polymers and added ZnS nanoparticles. At low frequencies, localized or immobilized dipoles, which facilitate long-range migration, contributed to the high dielectric constant. The dipoles polarized due to sufficient time at these frequencies, increasing dielectric loss. During the polarization process, the movement or alignment of these dipoles (short-range migration) contributed to some loss, resulting in higher dielectric loss values at lower frequencies. The dielectric loss and tan***δ*** decreased at higher frequencies due to the relaxation process [41]. In this frequency range, the electric field changed direction before the dipoles could orient along the field, resulting in a frequency-independent behavior and lower dielectric loss values. Also, the values of dielectric loss and tanδ of these nanocomposites are higher than that of the pure PEMA/PVC.

#### 3.6.2 The dielectric modulus

**Fig. 14:**
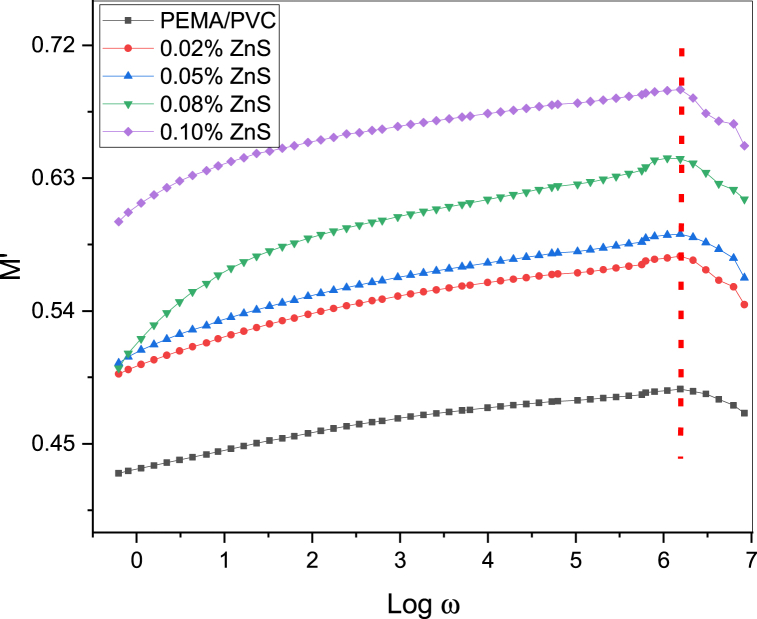
The plot between the real dielectric modulus ($M^{\prime }$) and log w of PEMA/PVC doped with ZnS.

**Fig. 15:**
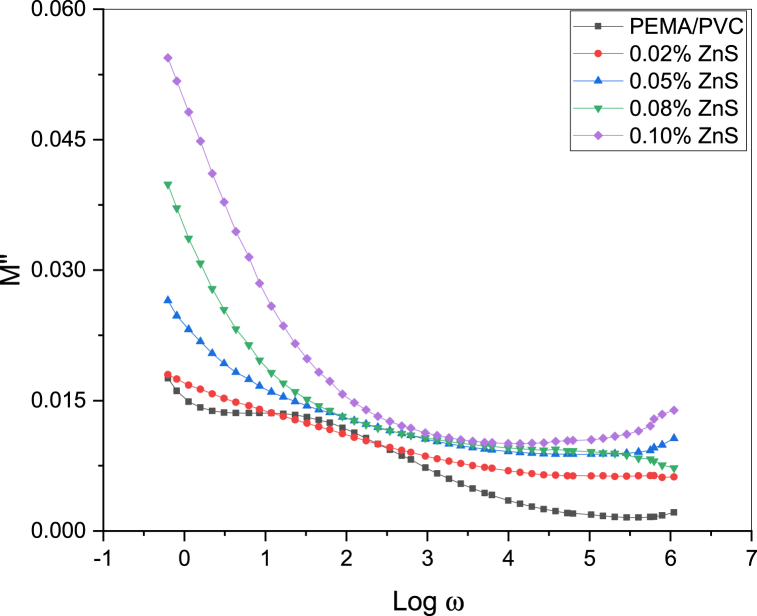
The plot between the imaginary dielectric modulus ($M^{\prime \prime }$) and log w of PEMA/PVC doped with ZnS.

Fig. 14 depicts the correlation between the real dielectric modulus $M^{\prime }$ and Log w at room temperature. The graph demonstrates a semi-linear increase in $M^{\prime }$ values from 0.43 for pure PEMA/PVC blend, and it reaches maximum values at near high frequencies (2.45 × 105 Hz) for the 0.1 % ZnS sample due to the relaxation process caused by higher electric polarization. These findings indicate that the dielectric modulus exhibits a reverse frequency behavior compared to the dielectric constant at room temperature. The imaginary part of the modulus ($M^{\prime \prime }$) as a function of frequency (Lof ω) is shown in Fig. 15. The analysis presented the maximum values of $M^{\prime \prime ,}$ suggesting the appearance of a dielectric relaxation at low frequencies, and it decreased with the increase in frequency.

## 5 Conclusion

Pure zinc sulfide (ZnS) nanoparticles were chemically synthesized and then added at various concentrations to a poly(ethyl methacrylate)/polyvinyl chloride (PEMA/PVC) blend to prepare a PEMA/PVC@ZnS nanocomposite by the casting technique. X-ray diffraction and FT-IR results confirmed the formation and interaction between ZnS nanoparticles and PEMA/PVC polymer blend. The X-ray spectra indicated an increase in the amorphous region. The TEM image of pure ZnS nanoparticles reveals almost rods and semi-spherical aggregates composed of nanometer-sized (∼14.6±2 nm) which agrees with the XRD data. The UV–Visible spectra showed a distinct absorption band at 432 nm attributed to the ZnS surface plasmon resonance. Optical parameters such as optical band gaps, the extinction coefficient (k), refractive index (n), dielectric constants (*ε*′ and *ε*''), and optical conductivity (σ_(opt._) were studied in detail. After adding ZnS, the values of the optical band gap energies decreased for both direct and forbidden transitions. PL displays less significant sensitivity for pure PEMA/PVC, while a strong emission peak at 442 nm, exhibiting a clear excitonic emission feature, was observed in PEMA/PVC@ZnS nanocomposite films finding provides compelling evidence for the interaction between ZnS and PEMA/PVC blend. From AC measurement, the dielectric permittivity and dielectric modulus values were increased with increasing ZnS contents. Both dielectric loss and tanδ values were higher than pure PEMA/PVC, while $M^{\prime }$ increased to a maximum at 2.45 × 10^5^ Hz due to higher electric polarization. Dielectric relaxation appeared at low frequencies, as indicated by the maximum values of $M^{\prime \prime }$, decreasing with increasing frequency The PEMA/PVC@ZnS films exhibit outstanding optical and dielectric properties making them appropriate for a range of electrical applications.

## Data availability

Data are available on request to the authors.

## Funding

No funding was obtained for this study.

## Additional information

No additional information is available for this paper.

## CRediT authorship contribution statement

**Nuha Al-Harbi:** Conceptualization, Data curation, Formal analysis, Funding acquisition, Investigation, Methodology, Resources, Supervision, Writing – original draft, Writing – review & editing.

## Declaration of competing interest

The authors declare that they have no known competing financial interests or personal relationships that could have appeared to influence the work reported in this paper.
